# A phenotype driven integrative framework uncovers molecular mechanisms of a rare hereditary thrombophilia

**DOI:** 10.1371/journal.pone.0284084

**Published:** 2023-04-25

**Authors:** Noël Malod-Dognin, Gaia Ceddia, Maja Gvozdenov, Branko Tomić, Sofija Dunjić Manevski, Valentina Djordjević, Nataša Pržulj

**Affiliations:** 1 Barcelona Supercomputing Center (BSC), Barcelona, Spain; 2 Department of Computer Science, University College London, London, United Kingdom; 3 Institute of Molecular Genetics and Genetic Engineering (IMGGE), University of Belgrade, Belgrade, Serbia; 4 ICREA, Barcelona, Spain; Mohammed VI Polytechnic University, MOROCCO

## Abstract

Antithrombin resistance is a rare subtype of hereditary thrombophilia caused by prothrombin gene variants, leading to thrombotic disorders. Recently, the Prothrombin Belgrade variant has been reported as a specific variant that leads to antithrombin resistance in two Serbian families with thrombosis. However, due to clinical data scarcity and the inapplicability of traditional genome-wide association studies (GWAS), a broader perspective on molecular and phenotypic mechanisms associated with the Prothrombin Belgrade variant is yet to be uncovered. Here, we propose an integrative framework to address the lack of genomic samples and support the genomic signal from the full genome sequences of five heterozygous subjects by integrating it with subjects’ phenotypes and the genes’ molecular interactions. Our goal is to identify candidate thrombophilia-related genes for which our subjects possess germline variants by focusing on the resulting gene clusters of our integrative framework. We applied a Non-negative Matrix Tri-Factorization-based method to simultaneously integrate different data sources, taking into account the observed phenotypes. In other words, our data-integration framework reveals gene clusters involved with this rare disease by fusing different datasets. Our results are in concordance with the current literature about antithrombin resistance. We also found candidate disease-related genes that need to be further investigated. CD320, RTEL1, UCP2, APOA5 and PROZ participate in healthy-specific or disease-specific subnetworks involving thrombophilia-annotated genes and are related to general thrombophilia mechanisms according to the literature. Moreover, the ADRA2A and TBXA2R subnetworks analysis suggested that their variants may have a protective effect due to their connection with decreased platelet activation. The results show that our method can give insights into antithrombin resistance even if a small amount of genetic data is available. Our framework is also customizable, meaning that it applies to any other rare disease.

## Introduction

Familial thrombophilia (increased risk of developing thrombosis) is a multifactorial and polygenic disorder in which gene-gene and gene-environmental interactions contribute to complex phenotype manifestations. It is a common pathology underlying ischemic stroke, venous thromboembolism, ischemic heart disease and pregnancy loss [1–3]. Out of all deaths per day worldwide, one in four is caused by thrombosis, making it a leading global cause of death and disability [4]. Despite many clinical studies, the complex mechanisms of thrombophilia have not yet been elucidated [1, 4]. Considering the impact of thrombosis as a global cause of death, it is crucial to understand the mechanisms related to thrombophilia to improve the diagnosis, prevention and treatment further.

In this study, we focus on a rare subtype of hereditary thrombophilia, antithrombin resistance. Antithrombin resistance is caused by variants in the prothrombin gene (F2) located in the antithrombin binding region of the protein. The impaired thrombin inhibition by antithrombin leads to thrombotic disorders [5]. The Prothrombin Belgrade variant (c.1787G>A, p. Arg596Gln) is one of the antithrombin resistance-causing variants and it was detected for the first time in subjects from the Serbian population [6].

While genome-wide association studies (GWAS) have already demonstrated their effectiveness in finding disease-associated variants for common diseases, these approaches are not suited to rare diseases due to the heterogeneity and rarity of genetic data [7, 8]. On the other hand, it has been proven that Non-negative Matrix Tri-Factorization (NMTF) can deal with a low sample size by integrating prior knowledge into the small datasets [9]. NMTF-based data integration methodologies allow for combing different types of biological and medical data to provide a comprehensive view of biological systems [10]. Here, we propose an NMTF-based data integration (fusion) method that leverages different data sources to get molecular insights into antithrombin resistance. In other words, by applying our integrative framework to identify novel disease-related genes for antithrombin resistance, we can overcome the lack of genetic samples and make concrete progress toward possible treatments.

**Fig 1:**
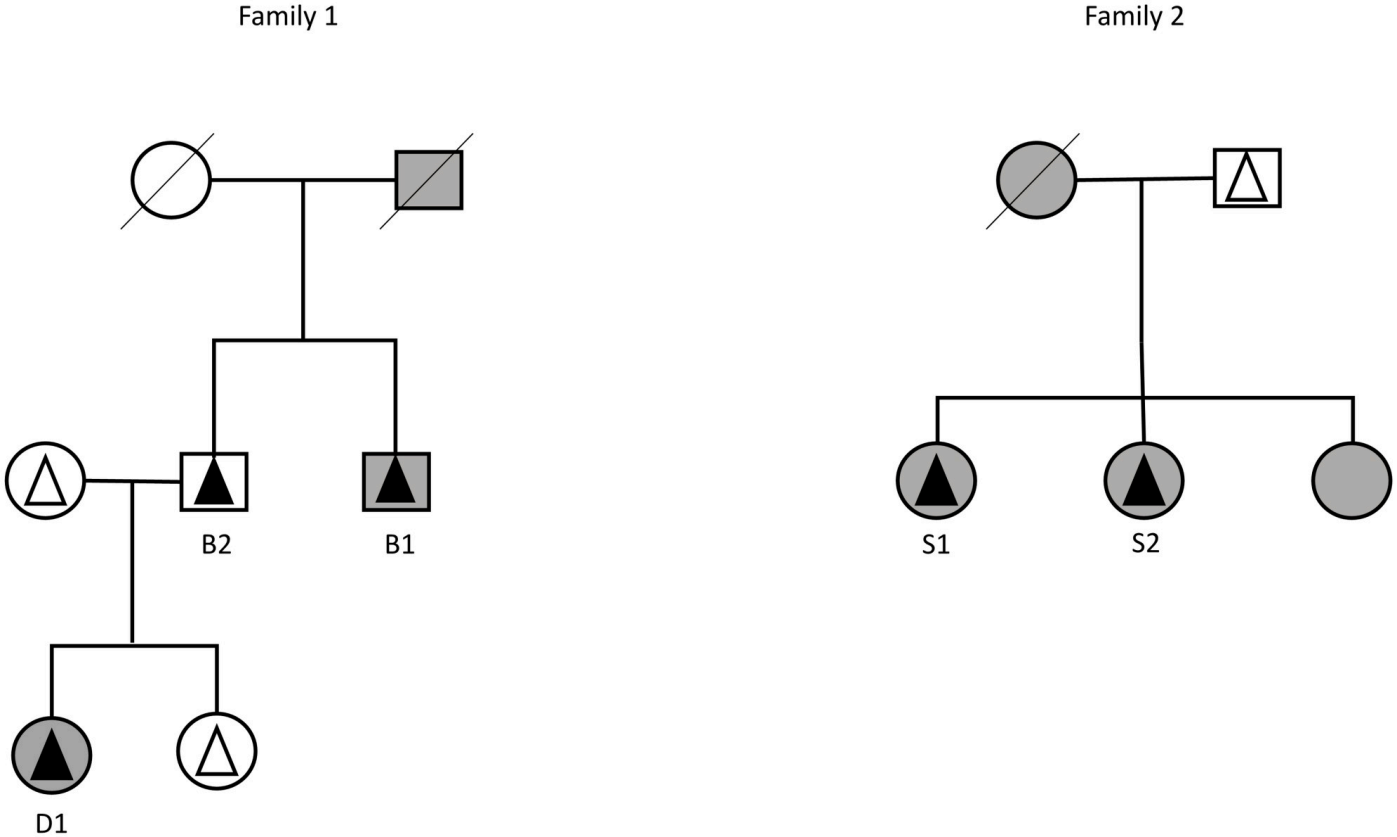
Observed phenotypes. Squares stand for men and circles stand for women. The triangles mark the tested family members: black triangles—heterozygous carriers of prothrombin Belgrade mutations, white triangles—non-carriers of the prothrombin Belgrade mutation. Thrombophilia: grey—subjects suffering from thrombosis, white—healthy subjects (possibly asymptomatic carriers). B1: brother 1, B2: brother 2, D1: daughter 1, S1: sister 1, S2: sister 2.

This study focuses on the full genome sequences of five heterozygous carriers (subjects) of the Prothrombin Belgrade variant from two Serbian families. These subjects have the following phenotypes. In family 1, the first brother, *B*_1_, developed recurrent venous thromboembolism, while the second brother, *B*_2_, is a healthy subject (asymptomatic carrier). Furthermore, the healthy brother has a daughter, *D*_1_, who also had recurrent thrombotic events. In family 2, two sisters, *S*_1_ and *S*_2_, experienced venous thromboembolism (illustrated in Fig 1).

To uncover genes for which our subjects possess germline variants (that we term “genes with variants”) associated with this rare subtype of hereditary thrombophilia, as well as the ones responsible for the healthy phenotype of brother *B*_2_, we integrate the germline variant profiles of the subjects with three omics molecular datasets (protein-protein interactions, gene co-expressions and genetic interactions) using Simultaneous Orthogonal Non-negative Matrix Tri-Factorization (SONMTF), detailed in Section Materials and methods. Also, we use the observed phenotypes as prior knowledge to enforce the subject stratification by separating healthy from diseased subjects in SONMTF. Then, we analyze the gene clusters resulting from SONMTF to find disease-related genes of antithrombin resistance. Our results demonstrate that integration and computational analysis of antithrombin resistance phenotypes and omics data using SONMTF has the potential to reveal novel insights into the biology of this rare genetic disease. Moreover, our framework’s straightforward applicability and effectiveness to a small number of genetic samples opens the door to its usage on other rare diseases.

## Materials and methods

First, we describe the omics data used in this study, i.e., the molecular networks, the germline variants and the biological annotation datasets. Next, we explain our new data fusion framework based on Non-negative Matrix Tri-Factorization (NMTF) to integrate all the considered heterogeneous omics data. Finally, we extract clusters of subjects and clusters of genes and do the enrichment analysis in biological annotations on the resulting clusters of genes (detailed below) from which we find gene clusters specific to the healthy subject (henceforth termed *healthy specific*) and gene clusters specific to the diseased subjects (henceforth termed *disease specific*) (i.e., those gene subnetworks containing genes that are mutated only in the healthy subject and genes that are mutated in all diseased subjects, respectively; see below on the definitions of molecular networks).

### Datasets

#### Molecular networks

We collect three large-scale molecular interaction datasets for humans. From BioGRID 3.5.176 [11], we collect the experimentally validated protein-protein interactions, PPIs (270,554 PPIs between 17,104 proteins). From COXPRESdb v7.3 [12], we collect the co-expressions between genes (thresholded to preserve only the top 1% strongest co-expressions, following the methodology from Malod-Dognin *et al.* [13], leading to 4,172,940 interactions between 25,063 genes). Finally, we collect the genetic interaction data from Rausher *et al.* [14] (391,527 genetic interactions between 16,249 genes). Each molecular dataset is modeled as a network in which nodes represent genes and in which two nodes are connected by an edge if the corresponding genes interact in the dataset. As needed by our NMTF-based data-integration model, each network is represented by its adjacency matrix, in which a value in a given row and column is equal to one if the corresponding genes interact and zero otherwise. These matrices are all reordered so that a given row and column, *i*, represents the same gene in all matrices. Following the approach of Malod-Dognin *et al.* [13], and because PPI is the most direct evidence that two genes can interact, we exclude from our study (by removing the corresponding rows and columns from all matrices) the genes that have no PPI information at all (i.e., whose corresponding rows and columns in the PPI matrix are all zeros). However, we did not further filtered-out genes based on their variant status. Thus, while our molecular network matrices all have the same set of 17,104 genes (with variants or not), their edge sets (the ones in the matrices) differ.

#### Germline variants

The whole-genome sequences of five heterozygous carriers (subjects) of the Prothrombin Belgrade variant from two Serbian families are captured by using BGI/MGI DNBSEQ sequencing technology, 40x read coverage (Complete Genomics) and assembled using the human reference genome build 37 (GRCh37). From the whole genome sequences, we extract the protein-coding germline variants of each subject using the NCBI Homo Sapiens Annotation Release 104 [15] and Complete Genomics Analysis Tools, CGA Tools (with default settings) to filter out variants based on confidence scores and read coverages. We consider all the non-synonymous variants that map to coding regions, namely: missense, nonsense, nonstop, misstart, frameshift, insert, insert+, delete, delete+ and disrupt. We did not apply filters to remove common variants since the ones that protect the asymptomatic brother may be common. We term “genes with variants” the genes for which our subjects possess such non-synonymous variants.

We verify that most of the genes with variants of the subjects are in the biological networks (about 79%, see Table 1). Also, to assess the relevance of these genes with variants to thrombophilia, we collect the variant-phenotype annotation dataset from DisGeNet v6.0 [16]. As detailed in S1 File, Section 3, we find that the genes with variants are highly related to thrombophilia and blood tests because 50.4% of their 492 associated phenotype annotations are either “Disease or Syndrome” annotations related to thrombophilia in PubMed publications, or “Laboratory Procedure” annotations related to blood tests, which is in accordance with thrombophilia-related tests and a risk of thrombosis (detailed in S1 File, Section 3).

**Table 1:** Germline variant dataset. For each subject (row), the table indicates the number of coding variants found in the subject’s genome (column “#Genes with variants”) and the number of these genes that are present in any of our molecular interaction datasets (column “#In the networks”).

Subject	#Genes with variants	#In the networks
Brother 1	5,807	4,574
Brother 2	5,913	4,649
Daughter 1	6,218	4,924
Sister 1	6,327	4,996
Sister 2	6,446	5,089

#### Biological annotations

From Gene Ontology [17], we collect the experimentally validated biological process (BP), molecular function (MF) and cellular component (CC) annotations of the genes. Furthermore, from Reactome [18], we collect the pathways (RP) and reactions (RR) that the genes participate in. Finally, from DisGeNet v6.0 [16], we collect a list of 83 thrombophilia-related genes, out of which 76 are present in the networks.

### Data integration framework

To uncover genes with variants that are relevant to our cases of thrombophilia, we propose a data integration framework based on Non-negative Matrix Tri-Factorization (NMTF) [19], a machine learning technique originally proposed for co-clustering and dimensionality reduction that was recently used for data fusion [13, 20–24].

**Fig 2:**
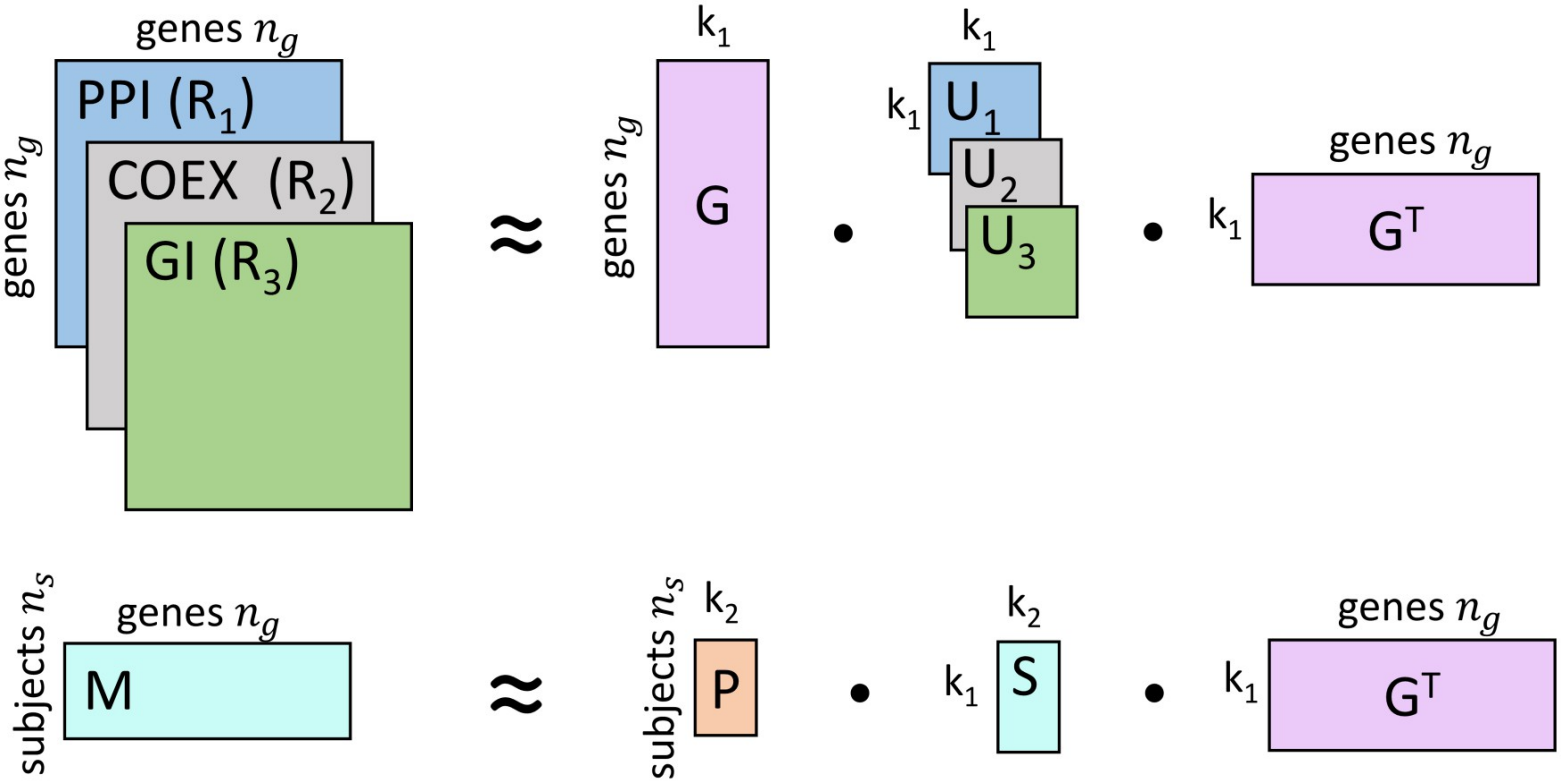
Our data-integration framework. We used the NMTF method to integrate four different data types: germline mutations of the five subjects presented in $M^{n_{s}\times n_{g}}$, protein-protein interactions (PPIs) from BioGRID presented in $R_{1}^{n_{g}\times n_{g}}$, coexpressions (COEXs) from COXPRESdb presented in $R_{2}^{n_{g}\times n_{g}}$ and genetic interactions (GIs) from [14] presented in $R_{3}^{n_{g}\times n_{g}}$. *M* is simultaneously decomposed into the product of three lower dimensional matrix factors, *P*, *S* and *G*^*T*^; molecular networks, *R*_*i*_, are simultaneously decomposed into the product of three factors, *G*, *U*_*i*_ and *G*^*T*^, as detailed in Materials and methods. *k*_1_ and *k*_2_ are the gene and the patient clusters, respectively.

In our framework, illustrated in Fig 2, we consider the germline variant profiles of five subjects and also three molecular networks. Subjects and genes are related to each other by germline variant profiles, constructed for *n*_*s*_ subjects over *n*_*g*_ genes and captured in the relation matrix, $M^{n_{s}\times n_{g}}$. Its entries are binary values, with *M*[*s*][*g*] = 1 if gene *g* is found to be mutated in subject *s*, and zero otherwise. Molecular networks, PPI, COEX and GI, are represented by their adjacency matrices, $R_{1}^{n_{g}\times n_{g}}$, $R_{2}^{n_{g}\times n_{g}}$ and $R_{3}^{n_{g}\times n_{g}}$, respectively. Their entries are binary values, with *R*_1_[*u*][*v*] = 1, *R*_2_[*u*][*v*] = 1, *R*_3_[*u*][*v*] = 1 if genes *u* and *v* interact in network PPI, COEX and GI, respectively, and zero otherwise. All these matrices are simultaneously decomposed as products of three matrix factors as *M* ≈ *P* ⋅ *S* ⋅ *G*^T^ and *R*_*i*_ ≈ *G* ⋅ *U*_*i*_ ⋅ *G*^T^ for 1 ≤ *i* ≤ 3, where $P^{n_{s}\times k_{2}}$ is interpreted as the *cluster membership indicator* matrix of subjects (grouping *n*_*s*_ subjects into *k*_2_ clusters), $G^{n_{g}\times k_{1}}$ is interpreted as the *cluster membership indicator* matrix of genes (grouping *n*_*g*_ genes into *k*_1_ clusters) that is shared across all decompositions and hence allows learning from all data, $S^{k_{1}\times k_{2}}$ is interpreted as the compressed representation of the molecular profiles (that indicates how the *k*_2_ clusters of subjects relates to the *k*_1_ clusters of genes in *M*), and $U_{i}^{k_{1}\times k_{1}}$ is interpreted as the compressed representation of each molecular network (that indicates how the *k*_1_ clusters of genes relate to each other in each molecular network). We also enforce the subject stratification (i.e., force the diseased subjects and the healthy subject to be in different clusters) by fixing the matrix factor, *P* (detailed in S1 File, Section 2). In other words, we take into account the observed phenotypes as prior knowledge to enforce the subject stratification with SONMTF. This leads to clusters of genes that better separate healthy-specific and disease-specific variants. We also demonstrate that our method is robust to data imbalance by comparing cluster stability of SONMTF runs, considering pairs of subjects as input (see S1 File, Section 2).

This decomposition is done by solving the following minimization function, approximately solving the NMTF problem for simultaneous factorization of matrices *M* and *R*_*i*_ into three matrix factors each, in which we have made matrix factors *P* and *G* orthogonal to minimize dependencies and provide a *hard* clustering interpretation of *P* and *G* [25] as mentioned above (hence the name Simultaneous Orthogonal Non-negative Matrix Tri-Factorization, SONMTF):

$$\begin{array}{c} \begin{array}{c} \underset{\text{P},\text{S},\text{G},{\text{U}}_{\text{i}}\geq 0}{\text{min}}||\text{M}-\text{PS}{\text{G}}^{\text{T}}{\left|\right|}_{\text{F}}^{2}\;\;+\;\;\sum_{\text{i}=1}^{3}\left|\right|{\text{R}}_{\text{i}}-\text{G}{\text{U}}_{\text{i}}{\text{G}}^{\text{T}}{\left|\right|}_{\text{F}}^{2},\\ \text{s.t.}\;{\text{P}}^{\text{T}}\text{P}\;=\;\text{I}\;\text{and}\;{\text{G}}^{\text{T}}\text{G}\;=\;\text{I}, \end{array} \end{array}$$ (1)

where F denotes the Frobenius norm. We heuristically minimize the above function using dedicated multiplicative update rules (see S1 File, Section 1).

### Extracting clusters and subnetworks

The resulting matrices, $P^{n_{s}\times k_{2}}$ and $G^{n_{g}\times k_{1}}$, are *cluster membership indicator* matrices for subjects and genes, respectively; based on their entries, *n*_*s*_ subjects are assigned to *k*_2_ subject clusters, *n*_*g*_ genes are assigned to *k*_1_ gene clusters, respectively. We extract subject and gene clusters from *P* and *G*, respectively, by using the hard clustering procedure as done by Brunet *et al.* [26]. In particular, for each row in *P*, we place subject s into cluster k if *P*[*s*][*k*] is the largest entry in row s. We apply the same procedure to extract gene clusters from *G*. Moreover, for each identified gene cluster, we create the corresponding *subnetwork* in which nodes represent genes in that specific cluster and in which two nodes are connected by an edge if the corresponding genes interact in any of the molecular interaction networks (since the edge set is different for each molecular interaction network).

### Enrichment in biological annotations

We identify the annotations that are significantly enriched in the clusters of genes as follows. The probability that an annotation is enriched in a cluster is computed using sampling without replacement strategy (also called the hypergeometric test) [27]:

p=1-∑i=0X-1(Ki)(M-KN-i)/(MN).

where N is the size of the cluster (only annotated genes from the cluster are taken into account), X is the number of genes in the cluster that are annotated with the annotation in question, M is the number of annotated genes in the network and K is the number of genes in the network that are annotated with the annotation in question. An annotation is considered to be significantly enriched if its enrichment p-value, after correction for multiple hypothesis testing [28], is lower than or equal to 5%. Then, we measure the quality of the clustering by the percentage of clusters having at least one enriched annotation.

### Ethics statement

This study was approved by the Ethic committee of the Institute of Molecular Genetics and Genetic Engineering, University of Belgrade (O-EO-004/2015/2). Written informed consent was obtained for all participants.

## Results

We report computational and biological results based on our data-integration framework. Computationally, we adapted our approach to consider subjects’ phenotypes as detailed in S1 File, Section 2. We used four different data types: germline variants, protein-protein interactions, co-expressions and genetic interactions. Each dataset is modeled as a network and integrated as detailed in Section Materials and methods. By leveraging the co-clustering interpretation of the SONMTF, which simultaneously decomposes molecular interactions (protein-protein interactions, co-expressions and genetic interactions) and germline variants datasets, we retrieved interesting gene clusters and the corresponding subnetworks as detailed in Section Extracting clusters and subnetworks (see Section Healthy-specific and disease-specific gene cluster analysis). We also use the PhD-SNPg method [29] to compare our results with a baseline approach that predicts the impact of germline variants. The results presented in S1 File, Section 3, show that the PhD-SNPg method identifies 1.01% of pathogenic variants in our datasets. In particular, two of the 44 reported gene clusters are significantly enriched in candidate pathogenic variants identified by PhD-SNPg (p-values <0.05).

### Healthy-specific and disease-specific gene cluster analysis

The outputs of the SONMTF method are clusters and subnetworks of genes (see Section Extracting clusters and subnetworks), that best separate diseased subjects from the healthy carrier (detailed in S1 File, Section 2). In particular, the resulting gene clusters are significantly enriched in GO biological processes (adjusted p-values <0.05), i.e., they are biologically coherent compared to random clusters (see S2 Fig in S1 File). In the following Sections, we focus on two clusters having the largest percentages of *healthy-specific variants* (genes that are mutated only in the asymptomatic carrier) and *disease-specific variants* (genes mutated in all diseased subjects, but not mutated in the healthy one), respectively. Moreover, we analyze two other clusters: one containing ADRA2A and TBXA2R, the only two thrombophilia-related genes mutated in the healthy subject and the other containing F2, the main antithrombin resistance-causing gene, which is mutated in all subjects. For each subnetwork, we highlight well-known thrombophilia genes present in the DisGeNet database.

#### Healthy-specific subnetwork

**Fig 3:**
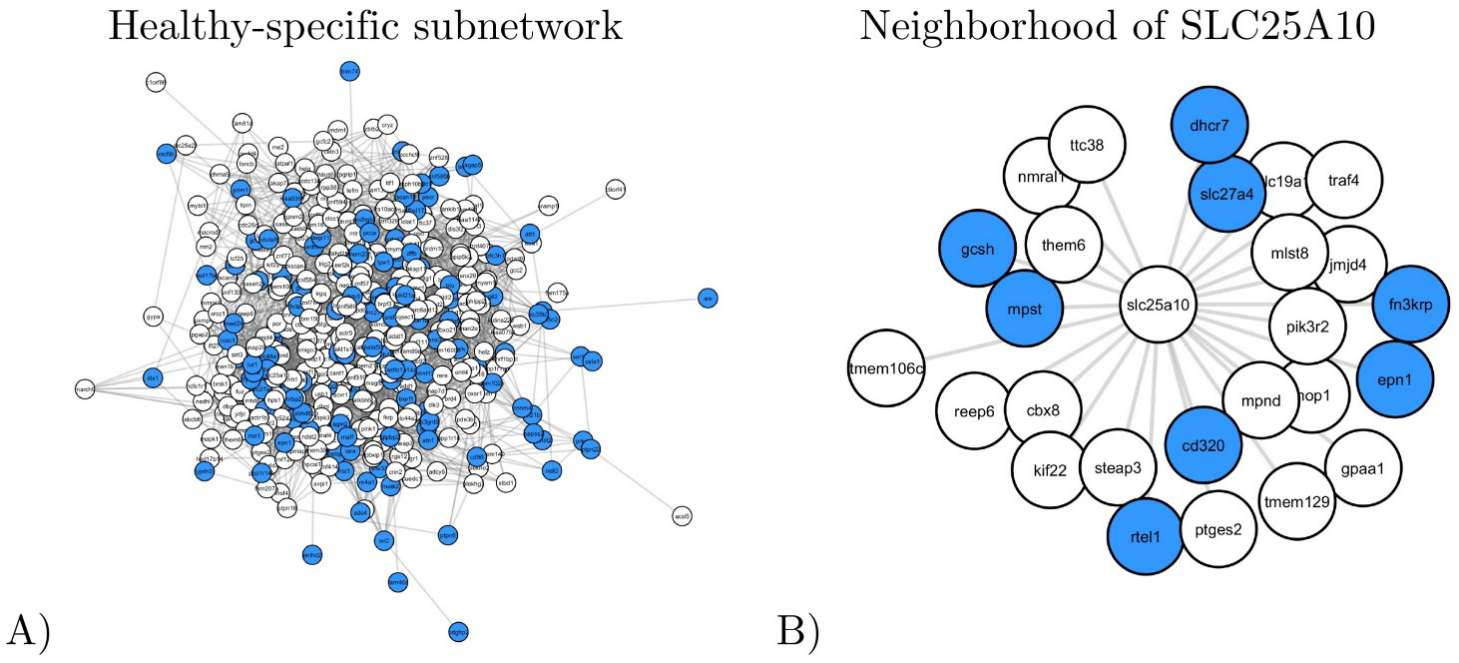
An illustration of the healthy-specific subnetwork. Nodes corresponding to the genes that have healthy-specific variants are colored in blue **A**: A spring embedding visualization of the subnetwork. **B**: The direct neighborhood of SLC25A10 in the subnetwork.

We focus on the cluster containing the highest percentage of healthy-specific variants, which corresponds to the *healthy-specific subnetwork* of 461 nodes (genes) and 3,115 edges. Illustrated in Fig 3, panel A, this subnetwork contains 29.1% of genes with healthy-specific variants (colored in blue) and does not contain any disease-specific variant genes (i.e., specific to diseased carriers). We hypothesize that this cluster contains specific variants that protect the healthy carrier from the disease.

Functionally, the healthy-specific subnetwork contains five protein-tyrosine phosphatases (PTPs) out of the 46 present in the entire dataset, and it is significantly enriched in “protein dephosphorylation” (GO:0006470). PTPs are known to be involved in the regulation of platelet activity, and thus, in thrombosis, validating our approach [30].

Within this subnetwork, SLC25A10 is the only gene known to be involved in thrombophilia, according to DisGeNet. SLC25A10 has 26 neighbors in the healthy-specific subnetwork (Fig 3, panel B), out of which eight are mutated only in the healthy subject: CD320, DHCR7, EPN1, FN3KRP, GCSH, MPST, RTEL1 and SLC27A4. While these genes are not associated with thrombophilia, we find literature evidence that suggests that some of these genes may be relevant. For instance, there is a possible association between variants in the RTEL1 gene and stroke risk in the Chinese population [31] while CD320 (transcobalamin 2 receptor) is known to be associated with hyperhomocysteinemia, which is a risk factor for cardiovascular disease [32].

#### Disease-specific subnetwork

**Fig 4:**
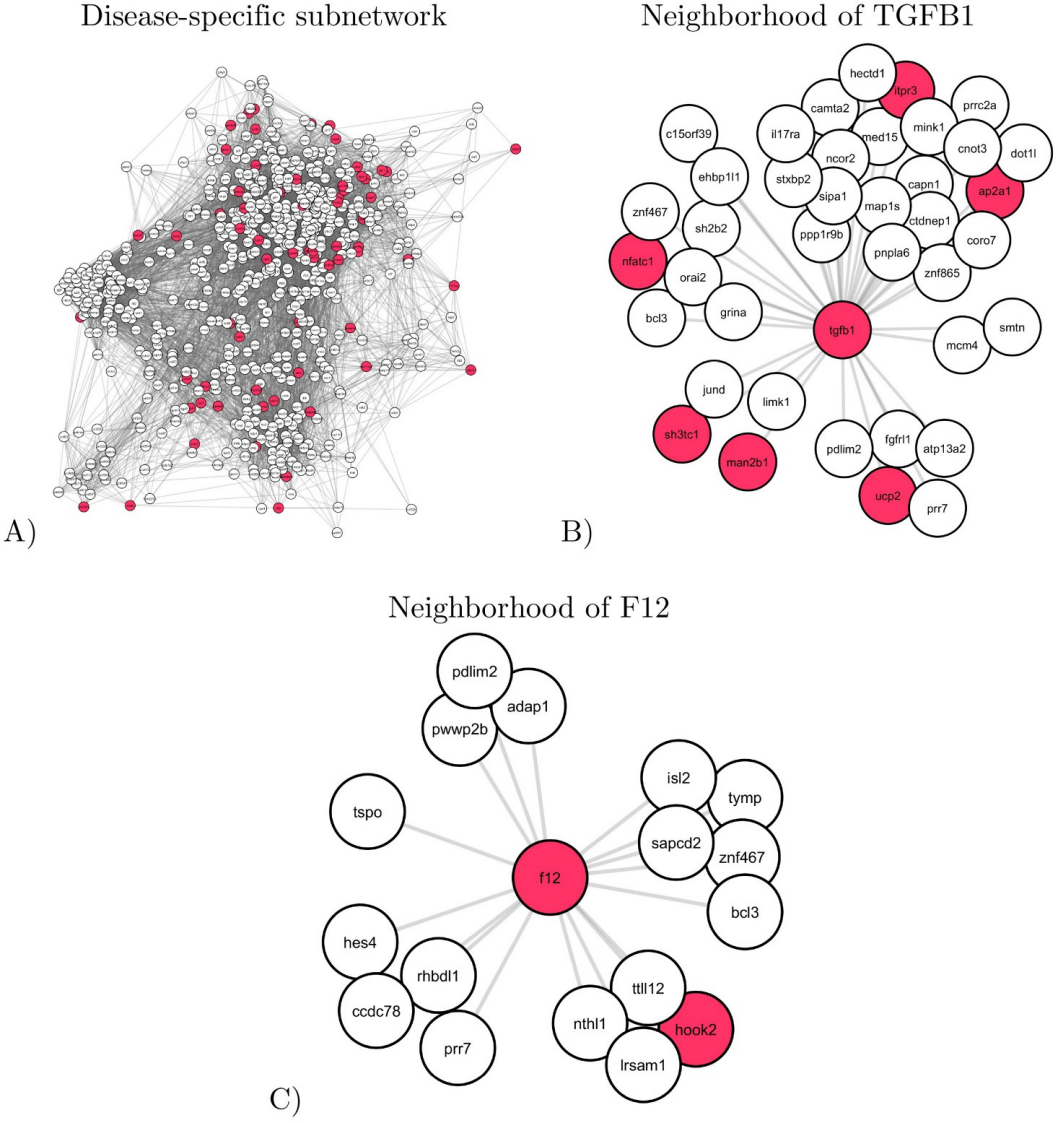
An illustration of the disease-specific subnetwork. Nodes corresponding to the genes that have disease-specific variants are colored in red. **A**: A spring embedding visualization of the subnetwork. **B** and **C**: The direct neighborhoods of TGFB1 (panel B) and F12 (panel C) in the subnetwork.

The cluster containing the largest percentage of genes with disease-specific variants corresponds to the subnetwork containing 695 nodes (genes) and 11,862 edges. Illustrated in Fig 4, panel A, this subnetwork contains 9.93% of genes with disease-specific variants (genes mutated in all diseased subjects, but not mutated in the healthy one, colored in red) and does not contain any gene with healthy-specific variant (genes that are mutated only in the healthy subject).

Functionally, the disease-specific subnetwork is significantly enriched in “Amplification of signal from unattached kinetochores via a MAD2 inhibitory signal” (R-HSA-141444) Reactome pathway. MAD2 is known to be involved in platelet production [33]; thus, a dysregulation of MAD2 may result in inappropriate platelet adhesion/activation and thrombosis [33]. The disease-specific subnetwork is also significantly enriched in “attachment of mitotic spindle microtubules to kinetochore” (GO:0051315), “metaphase plate congression” (GO:0051310) and “chromosome segregation” (GO:0007059) GO Biological Processes, all being part of the “cell cycle process” (GO:0022402). Wang *et al.* [34] showed that platelets could inhibit proliferation mainly through the arrest of the cell cycle and inhibition of DNA synthesis. Thus, the Reactome and GO enrichments detailed above may result from platelet alterations.

Moreover, the disease-specific subnetwork contains two thrombophilia-related genes present in the DisGeNet database, TGFB1 (transforming growth factor beta-1, TGF*β*-1) and F12 (coagulation factor XII), which are both disease-specific variants (Fig 4, panels B and C). TGFB1 gene regulates proliferation, maturation, differentiation, motility and apoptosis of cells [35]. It is also involved in the formation of blood vessels, wound healing, development of muscle tissue and body fat, inflammatory processes in the immune system and prevention of tumor growth [36, 37]. There are studies associating TGFB1 variants with heart disease, hypertension, myocardial infarction, and coronary artery disease [36, 38]. Though there is no direct interaction of proteins TGFB1 and thrombin, there are studies connecting their functions in immunity and hemostasis [39, 40]. Since all subjects in this study are heterozygous carriers of the Prothrombin Belgrade variant, it is possible that the TGFB1 variant could have an additive contribution to the clinical outcome of the subjects and lead to severe clinical manifestations.

F12 gene (coagulation factor XII) is essential for normal blood clotting [41]. An inactive form of F12 circulates in the bloodstream until it is activated. Upon activation, F12 interacts with the coagulation factor XI and with a plasma protein, prekallikrein. This process leads to increased permeability of blood vessel walls and inflammation [41]. In our study, we detect several different variants in F12 gene for symptomatic subjects. However, their relevance for the associated molecular mechanism of the Prothrombin Belgrade variant needs to be further investigated.

Among the direct neighbors of TGFB1 and F12 genes within the disease-specific subnetwork, we find disease-specific variants that are related to thrombophilia. For instance, among the neighbors of TGFB1, UCP2 gene (mitochondrial uncoupling protein-2) is mutated in diseased subjects. UCP2 is known to be associated with venous thrombosis [42].

#### ADRA2A and TBXA2R subnetwork

**Fig 5:**
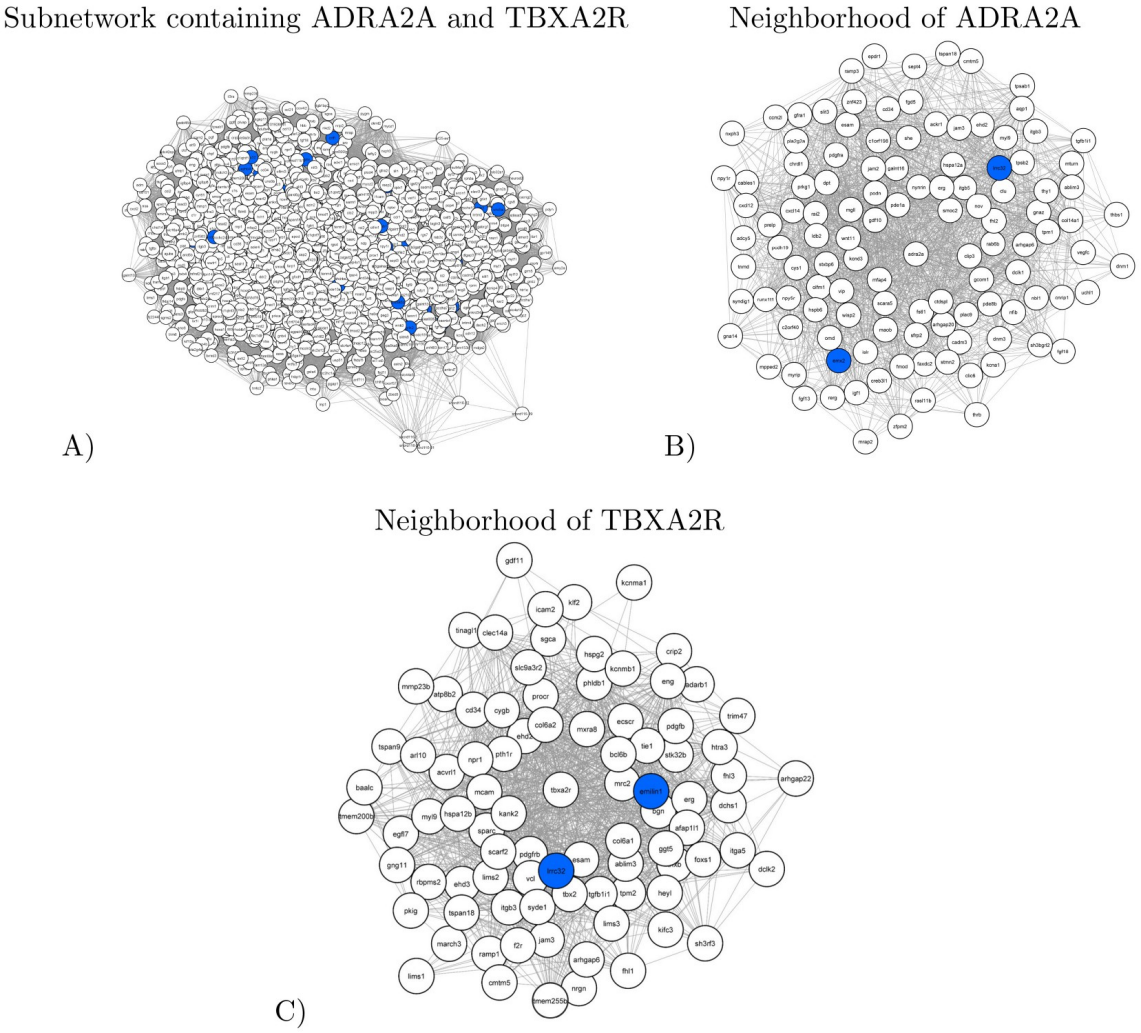
An illustration of the subnetwork containing ADRA2A and TBXA2R. Nodes corresponding to the genes that have healthy-specific variants are colored in blue. **A**: A spring embedding visualization of the subnetwork. **B** and **C**: The direct neighborhoods of ADRA2A (panel B) and TBXA2R (panel C) in the subnetwork.

The cluster containing ADRA2A and TBXA2R corresponds to the subnetwork of 1,591 nodes (genes) and 82,871 edges. Illustrated in Fig 5, panel A, the subnetwork contains 13 genes with healthy-specific variants (blue nodes in Fig 5). Functionally, the ADRA2A and TBXA2R subnetwork is significantly enriched in “Signaling by PDGF” (R-HSA-186797) Reactome pathway, namely the Platelet-derived Growth Factor (PDGF) signaling network. PDGF is a potent stimulator of growth and motility of connective tissue cells whose increase in protein expression has been associated with acute and chronic venous thrombosis [43]. Moreover, the ADRA2A and TBXA2R subnetwork is significantly enriched in“angiogenesis” (GO:0001525), “nervous system development” (GO:0007399) and “vasculogenesis” (GO:0001570) GO Biological Processes, all related to thrombophilia, or vascular disease.

ADRA2A and TBXA2R are thrombophilia-related genes mutated in the healthy subject. ADRA2A encodes for alpha-2-adrenergic receptors, expressed at the platelet surface and coupled with the G protein. The activation of these receptors leads to platelet activation and aggregation, which are essential components in thrombus formation [44]. This indicates the pivotal role of alpha-2-adrenergic receptors in hemostasis and suggests that variants of ADRA2A detected in the healthy subject could interact with the Prothrombin Belgrade variant. However, functional studies with mutated alpha-2-adrenergic receptors should be conducted to investigate whether those variants could represent gene modifiers responsible for the health status of the Prothrombin Belgrade variant carrier. TBXA2R encodes for the thromboxane A2 receptor, a member of the G protein-coupled receptor family and one of the most important mediators in the process of hemostasis. Thromboxane binding leads to receptor activation and induces platelet aggregation and activation. The thromboxane A2 receptor has two isoforms in humans, produced by alternative splicing of the primary transcript, which differs in the length of the cytoplasmic C-terminal end (15 amino acids for *α* and 79 amino acids for *β* isoform). The cytoplasmic tail is important for proper coupling of G protein and signal transduction, thus indicating that these two isoforms might have differential roles in the etiology of human diseases [45, 46]. In our study, the healthy subject has been shown to have variants in the TBXA2R gene, located in the cytoplasmic region of the *β* isoform of the thromboxane A2 receptor. So far, reported variants in this region are associated with the loss of receptor function and impaired thromboxane A2 agonist-induced platelet aggregation, resulting in bleeding disorders [45, 46]. We hypothesize that this variant could inhibit signal transduction, leading to decreased platelet aggregation, thus protecting the healthy subject from the occurrence of thrombosis. The effect of this variant on platelet function and its interaction with the Prothrombin Belgrade mechanism should be investigated in future studies.

For these reasons, the importance of analyzing ADRA2A and TBXA2R subnetwork lies in the fact that their variants could protect the healthy carrier from thrombosis disorders. Within this subnetwork, ADRA2A has 117 neighbors (Fig 5, panel B), out of which two are mutated only in the healthy subject: LRRC32 and EMX2. Interestingly, LRRC32 is also a neighbor of TBXA2R (Fig 5, panel C), which has 88 neighbors in total. LRRC32 is a key regulator of transforming growth factor beta, inducing a latent state of TGF-*β* in extracellular space [47]. This finding highlights the importance of TGFB1, as already mentioned in Section Disease-specific subnetwork.

#### F2 subnetwork

**Fig 6:**
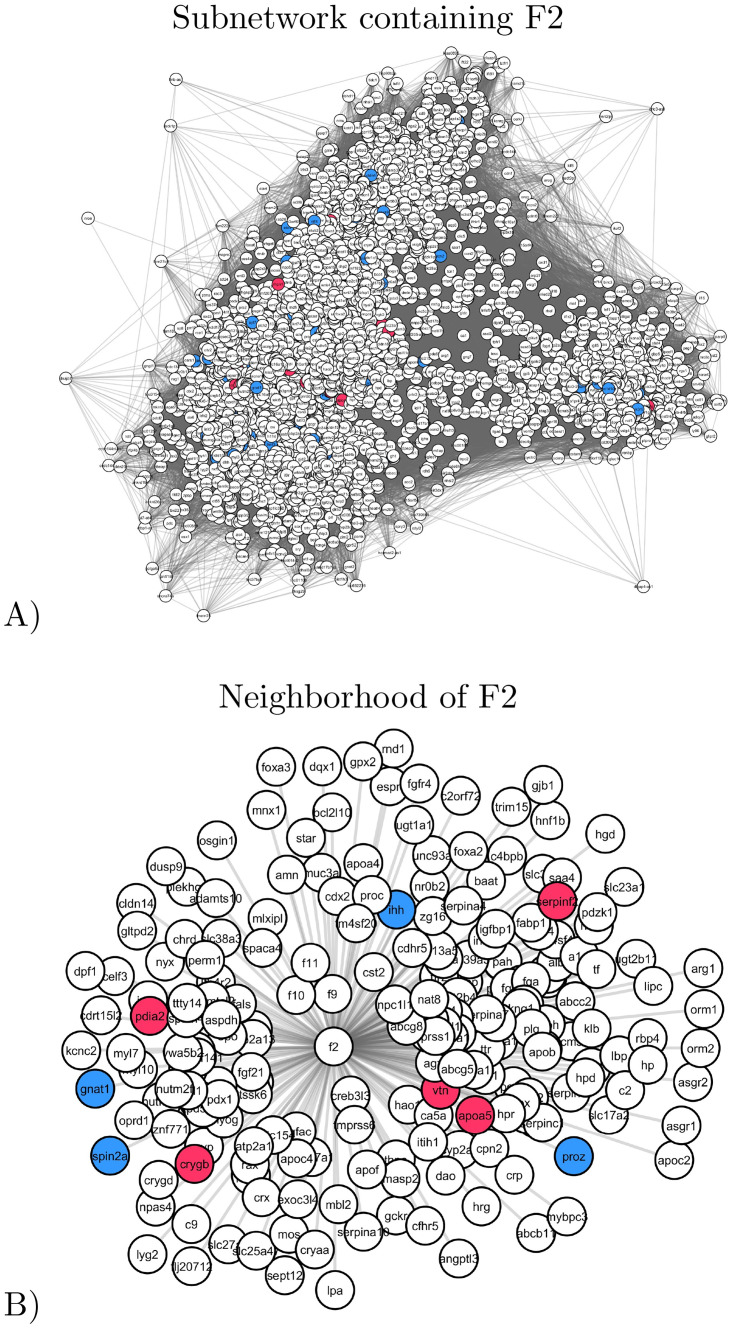
An illustration of the subnetwork containing F2, F5, F7, F9 and F11. Nodes corresponding to the genes that have disease-specific variants are colored in red and nodes corresponding to the genes that have healthy-specific variants are colored in blue. **A**: A spring embedding visualization of the subnetwork. **B**: The direct neighborhood of F2 in the subnetwork.

The subnetwork, containing F2, corresponds to a large network of 2,898 nodes and 178,748 edges. Illustrated in Fig 6, panel A, the F2 subnetwork contains 1.66% of genes with healthy-specific variants (genes mutated only in the healthy subject, in blue) and 0.76% of disease-specific variants (genes mutated in all diseased subjects but not mutated in the healthy one, colored in red).

Due to the large size of this subnetwork, functional enrichment analysis is not relevant. Instead, we focus on the thrombophilia-related genes. This subnetwork contains 23 thrombophilia-related genes, including many genes related to the activation of thrombin (F2, F5, F7, F9 and F11). Their variants are presented in S1 Table in S1 File. Within this subnetwork, F2 has 211 neighbors (Fig 6, panel B), our of which 5 have disease-specific variants, APOA5, CRYGB, PDIA2, SERPINF2 and VTN, and 4 have healthy-specific variants, GNAT1, IHH, PROZ and SPIN2A. While these genes are not related to thrombophilia in the DisGeNet database, we found literature evidence that they may be relevant to thrombophilia. For instance, APOA5 (apolipoprotein A5) is an important determinant of plasma triglyceride levels, which are associated with increased thrombosis risk [48]. CRYGB has been identified as a differentially expressed gene in Coronary Heart Disease [49]. PDIA2 is involved in the migration of human vascular smooth muscle cells [50]. SERPINF2 is an obvious candidate for venous thrombosis as it codes for a serpin protease inhibitor that acts as an inhibitor of plasmin [51]. Promoter variants of VTN are associated with vascular diseases [52]. The deletion of GNAT1 inhibits the development of retinal vascular pathology in early diabetic retinopathy [53]. IHH is involved in the Hedgehog (HH) pathway, whose functions include maintenance of the endothelial compartment and orchestrating revascularization upon vessel occlusion-induced ischemia [54]. PROZ (Protein Z) is associated with venous thrombosis [55].

## Discussion and conclusion

We proposed a data-integration method based on SONMTF to uncover molecular mechanisms behind a rare subtype of hereditary thrombophilia. Our integrative framework can cope with the lack of germline variant data, revealing interesting gene clusters. The results show that we can overcome the scarcity of samples by integrating omics network data with germline variant data. In other words, we can apply our integrative framework to any type of rare disease. Moreover, we applied our method to all coding variants present in our datasets without any filter, allowing for data-driven suggestions of candidate disease-related genes for the selected disease.

A possible limitation of this approach is that it requires phenotype information for each germline variant sample. As detailed in S1 File, our method is not completely unsupervised since it uses the observed phenotypes to constrain the grouping of the subjects. However, this is a small drawback compared to the benefit of uncovering molecular insights into rare diseases. Another potential limitation of our study is the construction of the co-expression network used as input into our NMTF-based data-integration model. In our study, we followed the approach from Malod-Dognin *et al.* [13], which yielded good results for the study of cancer. However, co-expression network construction and thresholding are still open questions with no gold standard solutions [56].

A possible future development is the incorporation of other prior information into the model, e.g., the zygosity of subjects or the type of coding variants. Another possible future development could be to assess how different co-expression measurements and thresholding strategies affect the ability of the framework to identify additional disease-related gene variants.

The analyses performed on the healthy-specific and disease-specific subnetworks obtained by our SONMF integrative framework show that several candidate disease-related genes for antithrombin resistance need further investigation. For instance, in the healthy-specific subnetwork, CD320, DHCR7, EPN1, FN3KRP, GCSH, MPST, RTEL1 and SLC27A4 may help to protect the healthy carrier from disease-specific variants. They are all connected to SLC25A10, the only thrombophilia-annotated gene in the subnetwork and two of them are validated in literature to have a role in thrombosis. The analysis of the disease-specific subnetwork revealed that the UCP2 gene might have an important role in thrombophilia as it is connected to TGFB1, a well-known thrombophilia gene present in the DisGeNet database. Moreover, the analysis of the F2 subnetwork revealed that APOA5, CRYGB, PDIA2, SERPINF2, VTN, GNAT1, IHH, PROZ and SPIN2A are mutated and directly connected to F2. We found literature evidence of the involvement of these genes in blood and vascular disorders; thus, they should be further investigated for their role in thrombophilia. Out of the 20 candidate genes that we newly proposed for thrombophilia, 17 of them have already been annotated for other diseases (detailed in S1 File, Section 4). Among them, MPST, SERPINF2, VTN and PROZ are annotated for bleeding disorders, providing interesting suggestions for candidate disease-related genes.

Our results are in concordance with the clinical study of Miljic et al. [57], which implies that platelets could participate in the mechanism of the Prothrombin Belgrade variant. They suggest that due to the antithrombin resistance, mutated thrombin is poorly inactivated, resulting in a prolonged period of platelet activation [57]. We hypothesize that variants in ADRA2A and TBXA2R genes might be associated with decreased platelet activation, compensating for the prolonged platelet activation caused by impaired thrombin inhibition in prothrombin Belgrade mutation carriers. This suggests that newly detected variants in these two genes might have a protective effect and represent gene modifiers in the Prothrombin Belgrade mechanism. However, further functional studies should be conducted to investigate their role in the Prothrombin Belgrade variant mechanism.

## Supporting information

S1 File Supplementary materials, figures and tables. (PDF)
