# Supplementary material for: A phenotype driven integrative framework uncovers molecular mechanisms of a rare hereditary thrombophilia
Source: PLoS One. 2023 Apr 25;18(4):e0284084. doi: 10.1371/journal.pone.0284084 (PMC10128975; doi:10.1371/journal.pone.0284084)
Supplement: S1 File — (PDF) [file pone.0284084.s001.pdf]

Supplementary Material for

A phenotype driven integrative framework uncovers molecular mechanisms of a rare hereditary thrombophilia

Noël Malod-Dognin,[1,2,¶] Gaia Ceddia,[1,¶] Maja Gvozdenov,[3] Branko Tomić,[3]
Sofija Dunjić Manevski,[3] Valentina Djordjević,[3]
Nataša Pržulj[1,2,4*]
[1]Barcelona Supercomputing Center (BSC), Barcelona, Spain.
[2]Department of Computer Science, University College London, United Kingdom.
[3]Institute of Molecular Genetics and Genetic Engineering (IMGGE), University of Belgrade, Belgrade, Serbia.
[4]ICREA, Barcelona, Pg. Lluís Companys 23, 08010, Spain.
* E-mail: natasha@bsc.es
¶ These authors contributed equally

1 Multiplicative update rules

As presented in Section Materials and methods of the main paper, the Simultaneous Orthogonal Non-negative Matrix Tri-Factorization, SONMTF, can be formulated as the following minimization problem:

$$\min_{P,S,G,U_i \geq 0} f(P,S,G,U_i) = \min_{P,S,G,U_i \geq 0} ||M - PSG^{\mathrm{T}}||_F^2 \quad + \quad \sum_{i=1}^{3} ||R_i - GU_iG^{\mathrm{T}}||_F^2,$$
$$\text{s.t. } P^{\mathrm{T}}P \ = \ I \text{ and } G^{\mathrm{T}}G \ = \ I,$$

where F denotes the Frobenius norm, M is a matrix containing the germline variant profiles of the subjects, R_1,R_2,R_3 represent the adjacency matrices of PPI, COEX and GI molecular networks, respectively, P is a matrix relating n_s subjects to n_g genes, S is interpreted as the compressed representation of the molecular profiles, G is interpreted as the cluster indicator matrix of genes, and U_i is interpreted as the compressed representation of each molecular network. Note that, as explained in the next section, P is a fixed matrix factor.

Following the semi-NMTF simplification [1] for a more computationally tractable solution, we remove the non-negativity constraint on $S, U_i \geq 0$. To solve the optimization problem, we derive the Karush-Kuhn-Tucker (KKT) conditions for our SONMTF as follows:

$$\frac{\partial f}{\partial G} = -M^T PS + GS^T P^T PS + \sum_i ((-2R_i^T GU_i + R_i GU_i^T) + 2(GU_i G^T GU_i^T + GU_i^T G^T GU_i)) - \eta = 0,$$
$$\frac{\partial f}{\partial S} = -P^T MG + P^T PSG^T G = 0,$$
$$\frac{\partial f}{\partial U_i} = -G^T R_i G + G^T GU_i G^T G = 0,$$
$$\eta, G \geq 0,$$
$$\eta \odot G = 0,$$

where $\odot$ is the Hadamard (element wise) product and matrix η is the dual variable for the primal constraint $G \geq 0$. Because adjacency matrices R_i are symmetric, therefore matrices U_i are

symmetric, too. For U_i and S, we have closed formulas:

$$\begin{aligned} U_i &= (G^T G)(G^T R_I G)(G^T G)^{-1} \\ S &= (P^T P)^{-1}(P^T M G)(G^T G)^{-1}. \end{aligned} \tag{1}$$

As explained in [2], we derive the following multiplicative update rule to solve the KKT conditions above:

$$G_{ij} \leftarrow G_{ij} \sqrt{\frac{(M^T PS)^+_{ij} + G(S^T P^T PS)^-_{ij} + \sum_i (R_i G U_i)^-_{ij} + (G(U_i G^T G U_i)^+)_{ij}}{(M^T PS)^-_{ij} + G(S^T P^T PS)^+_{ij} + \sum_i (R_i G U_i)^+_{ij} + (G(U_i G^T G U_i)^-)_{ij}}}. \tag{2}$$

We start from an initial solution, G_{init}, and iteratively use Equations (1) and (2) to compute new matrix factors U_i, S and G until convergence. To generate initial G_{init}, we use the Singular Value Decomposition based strategy [3]. This strategy makes the solver deterministic and also reduces the number of iterations that are needed to achieve convergence [3].

We measure the quality of the factorization by sum of the relative square errors (RSE) between the decomposed matrices and the corresponding decompositions:

$$RSE = \frac{||M - PSG^T||_F^2}{||M||_F^2} + \frac{\sum_i ||R_i - GU_i G^T||_F^2}{\sum_i ||R_i||_F^2}.$$

In our implementation, the iterative solver stops after 1000 iterations, the value for which the RSE of the decomposition is not decreasing anymore.

2 Subject stratification and gene clusters

In the main paper, we present the generic SONMTF framework used for integrating germline variants, protein-protein interactions, co-expressions and genetic interaction data. The outputs of the SONMTF algorithm are interesting gene clusters that take into account the phenotypic differences between diseased subjects and the healthy carrier. For this reason, in the first run of our data-integration framework, we set the number of subject clusters to two. By default, solving SONMTF leads to a subject stratification (from matrix factor P, see Section Materials and

methods of the main paper) that is best supported by the variant profiles of the subjects and by the considered molecular networks. In our case, subjects are grouped according to their family relationships, which is expected (see Figure 1, panel A). To account for the observed phenotypes of the subjects, it is possible to enforce the subject stratification (i.e., to force the diseased subjects to be in the same cluster and the healthy subject to be in a different cluster) by fixing matrix factor P (see Figure 1, panel B). For sanity check, for each of the two runs (when fixing or not the subject stratification), we extract the corresponding clusters of genes and measure their biological coherence by the percentage of them that are significantly enriched in at least one biological annotation. As presented in Figure 2, while forcing the subject stratification slightly reduces the functional enrichment of the obtained clusters of genes, our clusterings are highly biologically coherent.

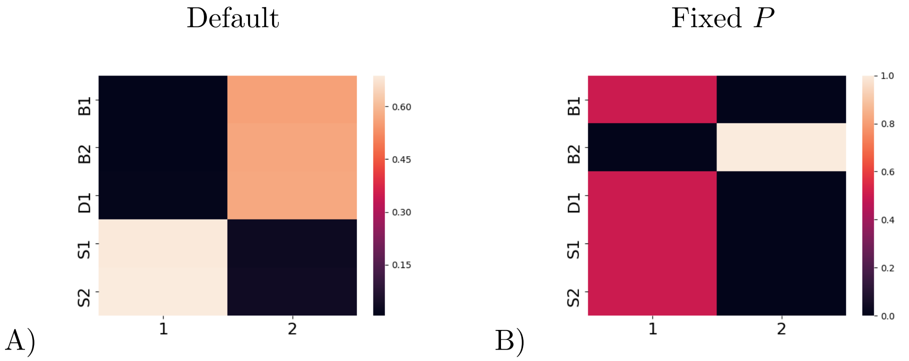

Figure S 1: **Cluster indicator matrices for the subjects**. **A:** The cluster indicator matrix, P, that is obtained when solving the default SONMTF (see Section Methods of main paper). It groups subjects B1, B2, and D1 into cluster 1, and subjects S1 and S2 into cluster 2. **B:** The cluster indicator matrix, P, that is kept fixed in order to group together the diseased subjects (B1, D1, S1, and S2) into cluster 1, and the healthy subject (B2) into cluster 2.

On the one hand, the clusters of genes that are obtained with the default solver lead to variant profiles (percentages of genes with variants per cluster) that are very similar across subjects (Figure 3, panel A), while the clusters of genes that are obtained when fixing P lead to variant profiles that are different for healthy and diseased subjects (Figure 3, panel B). Importantly, fixing P leads to clusters of genes that better separate healthy and disease-specific variants (Figure 3, panels C and D).

To test the robustness of our method to data imbalance, we make pairwise runs of our SON-

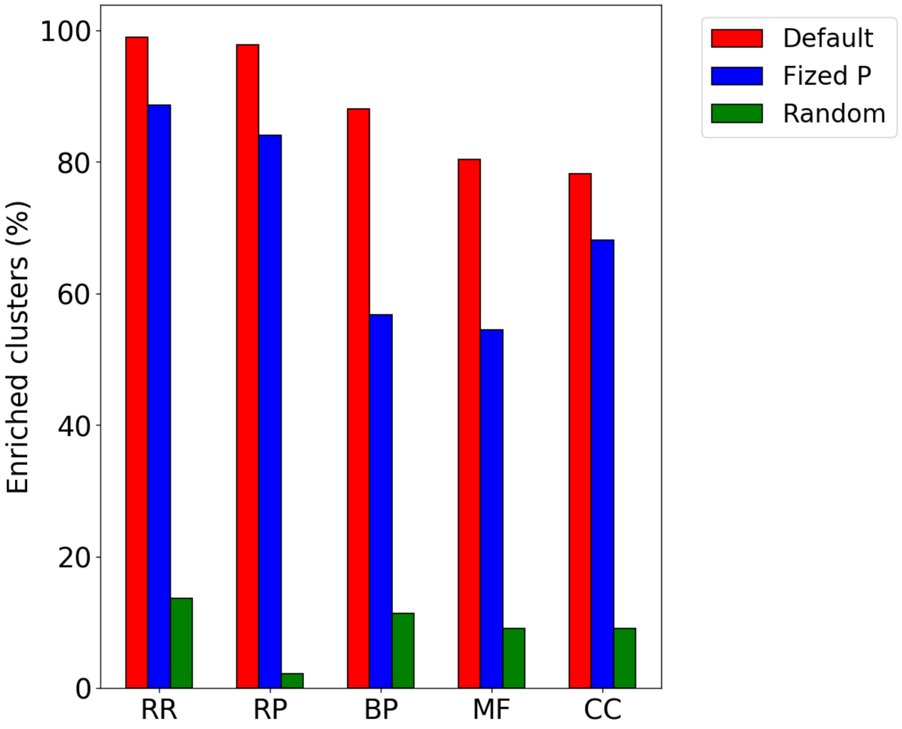

Figure S 2: **Cluster enrichment analysis**. For the clusterings that are obtained by using the default SONMTF (in red), by fixing the subject stratification (in blue) or by considering random clusters (in green) the bars present the percentage of the clusters that are significantly enriched in at least one biological annotation (see Section Enrichment in biological annotations) using Reactome Reaction (RR), Reactome Pathway (RP), gene ontology Biological Process (BP), gene ontology Molecular Function (MF), and gene ontology Cellular Component (CC) annotations.

MTF data integration framework using pairs of patients, i.e., B1-B2, D1-B2, S1-B2 and S2-B2. All the corresponding gene clusters are in agreement with the ones obtained when using all five individuals together, with Rand indices ranging from 88.7% to 90.1%. All these large agreements are statistically significant, with permutation-based p-values (using 100,000 permutations) all smaller than 10^{-5}. We also checked these agreements at the individual cluster level. For example, 86.7% to 95.3% of the genes from the ADRA2A-TBXA2R cluster are also grouped together in the same cluster in the pairwise clusterings. Hence, our methodology and results are robust to the data imbalance.

	Mutated in subject				
Gene	B1	B2	D1	S1	S2
ALB					
APOE					
APOH	1	1		1	1
CPB2	1	1	1	1	1
ELF3					
F11					
F2	1	1	1	1	1
F5	1		1	1	
F7	1			1	
F9					
FGA					
FGB		1	1		
FGFR4	1	1	1	1	1
HRG	1	1	1	1	1
LPA	1	1	1	1	1
PLG			1	1	
PROC					
REN					
SERPINA10		1	1	1	1
SERPINB2				1	1
SERPINC1					
SERPIND1				1	
STAT4					

Table S 1: **Thrombophilia related genes in cluster 19.** The table indicates if a given gene (row) is mutated in a given subject (column).

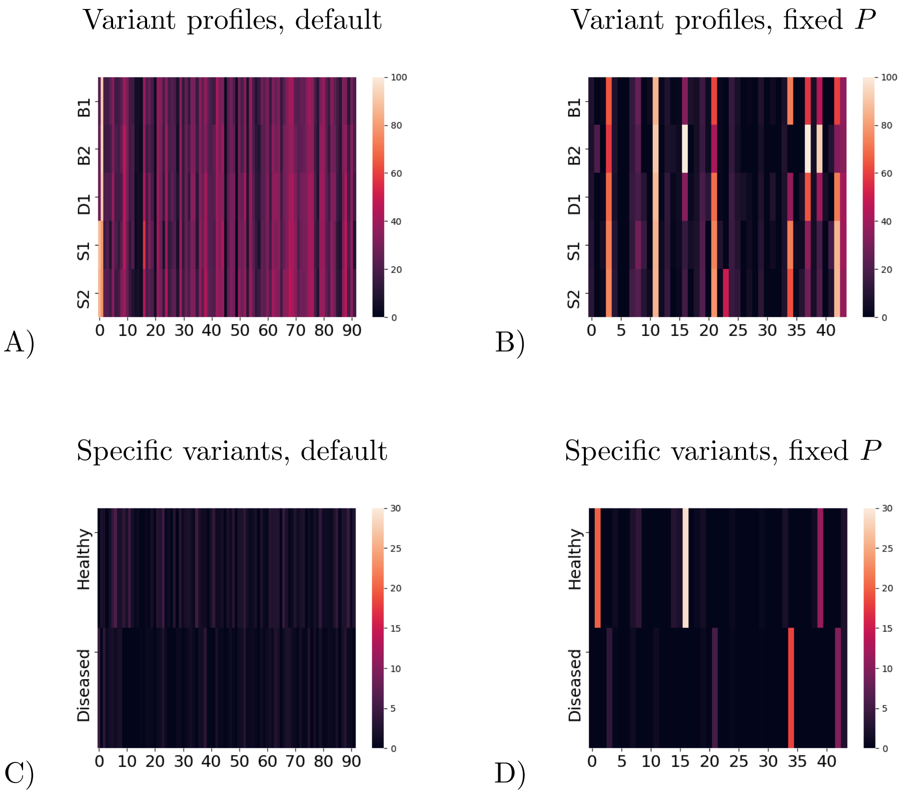

Figure S 3: **Cluster indicator matrices for the subjects**. **A:** variant profiles (percentages of genes with variants per clusters) that are obtained when solving the default SONMTF. **B** shows the same as (A), but for the clusters that are obtained when fixing P. **C:** For the clusters that are obtained when solving the default SONMTF, the first row indicates the profiles present only in the healthy subject. The second row reports the profiles present only in the diseased subjects. **D:** shows the same, but for the clusters that are obtained when fixing P.

3 Analysis of the germline variants

As a sanity check, we assess the relevance of the genes with variants of our five subjects with thrombophilia by investigating their known associated phenotypes in DisGeNet v6.0 [4]. These genes with variants are annotated with 492 phenotype annotations. In particular, the majority of the identified phenotypes are classified as "Laboratory Procedure" semantic type (123 phenotypes, out of 492), which are all related to blood tests, e.g., Blood Protein Measurement, Corpuscular Hemoglobin concentration Mean and Triglycerides Measurement. We further analyze the phenotypes classified as "Disease or Syndrome" by performing a systematic literature search in PubMed database [5]. We automatically retrieve the number of scientific publications that associate each

phenotype to thrombophilia by searching for co-occurrences between the two in PubMed. We call thrombophilia-related phenotypes those ones that co-occur at least one time with thrombophilia. We find that our gene variants are associated with 125 thrombophilia-related phenotypes out of 492, e.g., Rheumatoid Arthritis, Diabetes Mellitus, Non-Insulin-Dependent and Leukemia Myelocytic Acute. These findings suggest that considered gene variants are highly related to blood tests and disorders (50.4% of the phenotype annotations found for our gene variants are either thrombophilia-related or associated with blood tests), which is in accordance with thrombophilia-related tests and risk of thrombosis. Indeed, both Rheumatoid Arthritis and Diabetes Mellitus are autoimmune diseases that are linked to an increased risk of venous thrombosis [6]. To assess the relevance of the observation that thrombophilia co-occurs with 125 of the “Disease or Syndrome” phenotypes that annotate the subjects’ genes with variants in PubMed articles, we also measure the co-occurrences between any two of these “Disease or Syndrome” phenotypes in PubMed articles. We find that thrombophilia co-occurs more with the phenotypes that annotate the subjects’ genes with variants in PubMed articles than 73% of the considered “Disease or Syndrome” phenotypes. This further suggests that the genes with variants of our five subjects are indeed related to thrombophilia.

Moreover, we analyze the impact of our germline variants using the PhD-SNPg method [7]. We use PhD-SNPg to extract pathogenic variants and compare these results with our findings. We find that only 173 out of the 17,104 genes considered in our study are predicted as pathogenic variants (p-values$<$ 0.05). Moreover, we compute a hypergeometric test to check if these pathogenic variants are over-represented in our reported clusters. The results show that the cluster containing F2 also contains 14 predicted pathogenic variants and F12/TGFB1 cluster contains 12 predicted pathogenic variants out of 695 (hypergeometric p-values$<$ 0.05). Instead, the disease-specific subnetwork contains six predicted pathogenic variants out of 461, and the healthy-specific subnetwork does not contain them, which is in accordance with its healthy specificity. Interestingly, PhD-SNPg predicted pathogenic variants are not annotated as thrombophilia genes.

4 Candidate genes overview

In the main manuscript, we find some candidate genes that need to be further investigated. However, the majority of these genes have already been annotated for other diseases (17 out of 20). We report in Table 2 their disease associations found using DisGeNet. Interestingly, some of the diseases associated with our candidate genes are related to blood conditions.

**Candidate gene**	**Disease**
CD320	Encephalitis, Fever
DHCR7	Smith-Lemli-Opitz Syndrome, Movement Disorders
FN3KRP	Diabetes, Endothelian dysfunction
GCSH	Nonketotic Hyperglicemia
MPST	Corpuscolar Hemoglobin Concetration Mean
RTEL1	Glioma
SCL27A4	Ichthyosis Prematurity Syndrome
UCP2	Diabetes
APOA5	Serum total cholesterol measurement, Triglycerides measurements
CRYGB	Crystalline cataract
GNAT1	Night Blindness
SERPINF2	Aortic Aneurysm, Cerebral Infarction
VTN	Blood Protein Measurement
IHH	Bradrydactyly, type A1
PROZ	Anemia Sichke Cell, Vaso-Occlusive Crisis
ADRA2A	Osteoporosis, Metabolic Diseases
TBXA2R	Asthma, Cerebral Infaction

Table S 2: **Thrombophilia candidate genes.** The table shows DisGeNet annotation for the candidate genes.

References

[1] Ding CH, Li T, Jordan MI. Convex and semi-nonnegative matrix factorizations. IEEE Transactions on Pattern Analysis and Machine Intelligence. 2008; 32(1):45–55.

[2] Pržulj N. Analyzing Network Data in Biology and Medicine: An Interdisciplinary Textbook for Biological, Medical and Computational Scientists. Cambridge University Press; 2019.

[3] Qiao H. New SVD based initialization strategy for non-negative matrix factorization. Pattern Recognition Letters. 2015; 63:71–77.

[4] Piñero J, Bravo À, Queralt-Rosinach N, Gutiérrez-Sacristán A, Deu-Pons J, Centeno E, et al. DisGeNET: a comprehensive platform integrating information on human disease-associated genes and variants. Nucleic Acids Research. 2016; p. gkw943.

[5] Geer LY, Marchler-Bauer A, Geer RC, Han L, He J, He S, et al. The NCBI biosystems database. Nucleic Acids Research. 2010; 38(suppl_1):D492–D496.

[6] Zöller B, Li X, Sundquist J, Sundquist K. Autoimmune diseases and venous thromboembolism: a review of the literature. American Journal of Cardiovascular Disease. 2012; 2(3):171.

[7] Capriotti E, Fariselli P. PhD-SNPg: a webserver and lightweight tool for scoring single nucleotide variants. Nucleic Acids Research. 2017; 45(W1):W247–W252.
